# Development and validation of the OSASH score to predict overall survival of hepatocellular carcinoma after surgical resection: a dual-institutional study

**DOI:** 10.1007/s00330-023-09725-7

**Published:** 2023-05-16

**Authors:** Hong Wei, Fangfang Fu, Hanyu Jiang, Yuanan Wu, Yun Qin, Huanhuan Wei, Ting Yang, Meiyun Wang, Bin Song

**Affiliations:** 1 Department of Radiology, West China Hospital, Sichuan University, No. 37, GUOXUE Alley, Chengdu, 610041 Sichuan China; 2 Department of Medical Imaging, Henan Provincial People’s Hospital, No. 7, WEIWU Road, Zhengzhou, 450003 Henan China; 3 Department of Medical Imaging, People’s Hospital of Zhengzhou University, Zhengzhou, Henan China; 4 Big Data Research Center, University of Electronic Science and Technology of China, Chengdu, Sichuan China; 5 Academy of Medical Sciences, People’s Hospital of Zhengzhou University, Zhengzhou, Henan China; 6 Department of Radiology, Sanya People’s Hospital, Sanya, Hainan China

**Keywords:** Carcinoma, hepatocellular, Prognosis, Mortality, Magnetic resonance imaging, Hepatectomy

## Abstract

**Objective:**

To develop and validate a risk score based on preoperative clinical-radiological parameters for predicting overall survival (OS) in patients undergoing surgical resection for hepatocellular carcinoma (HCC).

**Methods:**

From July 2010 to December 2021, consecutive patients with surgically-proven HCC who underwent preoperative contrast-enhanced MRI were retrospectively enrolled. A preoperative OS risk score was constructed in the training cohort using a Cox regression model and validated in a propensity score-matched internal validation cohort and an external validation cohort.

**Results:**

A total of 520 patients were enrolled, among whom 210, 210, and 100 patients were from the training, internal validation, and external validation cohorts, respectively. Independent predictors for OS included incomplete tumor “capsule,” mosaic architecture, tumor multiplicity, and serum alpha-fetoprotein, which were incorporated into the “OSASH score.” The C-index the OSASH score was 0.85, 0.81, and 0.62 in the training, internal, and external validation cohorts, respectively. Using 32 as the cutoff point, the OSASH score stratified patients into prognostically distinct low- and high-risk groups among all study cohorts and six subgroups (all *p* < 0.05). Furthermore, patients with BCLC stage B-C HCC and OSASH-low risk achieved comparable OS to that of patients with BCLC stage 0-A HCC and OSASH-high risk in the internal validation cohort (5-year OS rates, 74.7 vs. 77.8%; *p* = 0.964).

**Conclusion:**

The OSASH score may help predict OS in HCC patients undergoing hepatectomy and identify potential surgical candidates among those with BCLC stage B-C HCC.

**Clinical relevance statement:**

By incorporating three preoperative MRI features and serum AFP, the OSASH score may help predict postsurgical overall survival in patients with hepatocellular carcinoma and identify potential surgical candidates among those with BCLC stage B and C HCC.

**Key Points:**

*• The OSASH score incorporating three MRI features and serum AFP can be used to predict OS in HCC patients who received curative-intent hepatectomy.*

*• The score stratified patients into prognostically distinct low- and high-risk strata in all study cohorts and six subgroups.*

*• Among patients with BCLC stage B and C HCC, the score identified a subgroup of low-risk patients who achieved favorable outcomes after surgery.*

**Supplementary Information:**

The online version contains supplementary material available at 10.1007/s00330-023-09725-7.

## Introduction

Liver cancer is the sixth most lethal malignancy globally, and hepatocellular carcinoma (HCC) accounts for  ~ 90% of primary liver cancer cases [1, 2]. Individualized prognostication is critical for informing optimal patient care in HCC. To this end, several staging systems have been developed based on tumor burden (e.g., size, number, vascular invasion, and extrahepatic metastasis), liver function, and patient performance status [3–7]. Among them, the most widely used system is the Barcelona Clinic Liver Cancer (BCLC) system, which allows prognostication and subsequent treatment allocation [3]. Nonetheless, there is great survival heterogeneity in each risk subgroup [8, 9], shedding light on the unsatisfactory performance of current systems in profiling the comprehensive landscape of tumor aggressiveness and the unmet need for HCC prognostication refinement.

Hepatectomy is the backbone for curative-intent treatment in early-stage HCC, offering a 5-year survival of 35–70% [2]. Nevertheless, controversy still shrouds the surgical indications for HCC. In line with the BCLC system, Western practice guidelines restrict liver resection to patients with very early- to early-stage HCC [10, 11]. However, growing evidence showed more favorable outcomes for liver resection in selected patients with intermediate- to advanced-stage HCC in comparison to transarterial chemoembolization and systemic therapies [12–16], as incorporated into major Asian guidelines [7, 17–19]. In spite of this, data remain scarce regarding the optimal surgical candidates among patients with intermediate- to advanced-stage HCC.

Overall survival (OS) has been widely accepted as the most important endpoint in oncology and HCC research and is not subject to investigator bias. Encouraging results have been reported on the utility of gadoxetate disodium–enhanced magnetic resonance imaging (EOB-MRI) features for noninvasive prediction of worse OS in HCC patients after treatment [20–23]. Nevertheless, these studies were hampered by a small sample size (e.g., 120–376 patients) and lack of external validation. Additionally, there are limited data on the comparison between the EOB-MRI and extracellular contrast agent-enhanced MRI (ECA-MRI) in HCC prognostication, whilst the latter is a more available, cost-effective, and time-efficient examination with superior arterial phase quality [24]. Furthermore, to our knowledge, few attempts have been made to assess the capacity of MRI-based prognostic tools in informing surgical recommendations for patients with intermediate- to advanced-stage HCC.

Therefore, we aimed to develop and externally validate a risk score based on preoperative clinical-radiological data to predict OS in HCC patients after hepatectomy. Additionally, we sought to investigate whether this score could be used to stratify prognosis and identify patients with intermediate- to advanced-stage HCC who would potentially achieve favorable prognosis after surgery.

## Materials and methods

This retrospective dual-institutional study was approved by the institutional review boards of West China Hospital, Sichuan University (Chengdu, Sichuan, China) and Henan Provincial People’s Hospital (Zhengzhou, Henan, China). The requirements for informed consent were waived.

### Patients

From July 2015 to November 2020, consecutive patients who underwent preoperative contrast-enhanced EOB-MRI within 2 months before resection for HCC at institution 1 were retrospectively enrolled and constituted the training cohort (termed the “EOB-MRI cohort”). The inclusion criteria were (a) surgically proven HCC; (b) R0 resection (defined as the complete macroscopic removal of tumor with a negative microscopic margin); (c) absence of a previous history of HCC treatment; and (d) absence of any co-malignancy other than HCC. The exclusion criteria were (a) distant metastasis at preoperative work-ups; (b) ruptured HCC; (c) incomplete clinical or pathological data; (d) inadequate MR images for analysis; and (e) without follow-up information.

If the developed risk score incorporated HBP imaging features, internal validation would be performed in the EOB-MRI cohort; otherwise, internal validation would be performed in an independent ECA-MRI cohort to test the model’s generalizability in broader populations. Specifically, from July 2010 to December 2021, consecutive patients who underwent preoperative ECA-MRI within 2 months before resection for HCC at institution 1 following the same eligibility criteria were enrolled and constituted the “ECA-MRI cohort.” The ECA-MRI-based internal validation cohort was created using propensity score matching as detailed in the “Statistical analysis” section. The selection of these two MRI contrast agents was based on the clinicians’ recommendations and patients’ preferences.

From April 2014 to March 2019, consecutive patients who underwent EOB-MRI within 2 months prior to resection for HCC at institution 2 following the same eligibility criteria were retrospectively enrolled and formed the external validation cohort.

In all study cohorts, data including clinical information, laboratory indexes (i.e., aspartate aminotransferase, alanine aminotransferase, total bilirubin, albumin, platelet, prothrombin time [PT], the international normalized ratio [INR], alkaline phosphatase, gamma-glutamyl transferase, and alpha-fetoprotein [AFP]) within 1 month prior to surgery and histopathological results were recorded. Baseline laboratory data with  ≤ 5% missingness were imputed by medians, with 0.4% (2/520) of missing values for AFP, 0.2% (1/520) for PT, and 0.5% (2/420) for INR. Cirrhosis was diagnosed by the recommendations in the Clinical Practice Guidelines [25]. The albumin-bilirubin (ALBI) score was calculated using previously described approaches [26].

### MRI technique

MRI was performed with six 3.0-T systems and two 1.5-T systems. Liver MRI sequences included: T2-weighted imaging, diffusion-weighted imaging with apparent diffusion coefficient maps, T1-weighted in- and opposed-phase imaging, and T1-weighted dynamic imaging consisting of precontrast phase, late arterial phase, portal venous phase, delayed phase (ECA-MRI) or transitional phase (EOB-MRI), and HBP (EOB-MRI) images. Details on MRI protocols are provided in Supplementary Material 1 and Table S1.

### Image analysis

All deidentified MR images were transmitted to institution 1 and reviewed independently by two fellowship-trained abdominal radiologists (readers 1 and 2, with 7 and 10 years of experience in liver MRI, respectively) who were informed that all enrolled patients had HCC, but were unaware of the remaining clinicopathological and follow-up information. Any discrepancy in imaging interpretation was resolved by a senior abdominal radiologist (reader 3, with over 20 years of experience in liver MRI).

On a per-patient basis, the following features were evaluated: (a) imaging features related to tumor burden [10, 11, 27]; (b) presence or absence of major, ancillary, LR-TIV and LR-M features as defined by Liver Imaging Reporting and Data System (LI-RADS) version 2018 [27]; (c) presence or absence of other imaging features related to tumor biology or patient outcomes [28–31]; and (d) presence or absence of imaging features related to underlying liver diseases [32, 33] (Table 1). For multiple tumors, radiologic features of the largest tumor were recorded for analysis.

**Table 1:** All evaluated MRI features and the definitions

Feature	Definition
Tumor burden-related features	
Tumor multiplicity	Number of definite intrahepatic HCC lesions with characteristic enhancement pattern [10, 11]
Tumor size	See page 16–157 of Chapter 16 of LI-RADS v2018 CT/MRI Manual [27]
Bilobar involvement	Bilobar involvement of definite HCC on contrast-enhanced MRI
LI-RADS major features	
Nonrim APHE	See page 16–66 of Chapter 16 of LI-RADS v2018 CT/MRI Manual [27]
Nonperipheral "washout"	See page 16–138 of Chapter 16 of LI-RADS v2018 CT/MRI Manual [27]
Enhancing "capsule"	See page 16–187 of Chapter 16 of LI-RADS v2018 CT/MRI Manual [27]
LI-RADS ancillary features	
* Favoring malignancy in general, not HCC in particular*	
Corona enhancement	See page 16–265 of Chapter 16 of LI-RADS v2018 CT/MRI Manual [27]
Fat sparing in solid mass	See page 16–272 of Chapter 16 of LI-RADS v2018 CT/MRI Manual [27]
Diffusion restriction	See page 16–278 of Chapter 16 of LI-RADS v2018 CT/MRI Manual [27]
Mild-moderate T2 hyperintensity	See page 16–283 of Chapter 16 of LI-RADS v2018 CT/MRI Manual [27]
Iron sparing in solid mass	See page 16–289 of Chapter 16 of LI-RADS v2018 CT/MRI Manual [27]
TP hypointensity	See page 16–295 of Chapter 16 of LI-RADS v2018 CT/MRI Manual [27]
HBP hypointensity	See page 16–300 of Chapter 16 of LI-RADS v2018 CT/MRI Manual [27]
* Favoring HCC in particular*	
Nonenhancing "capsule"	See page 16–309 of Chapter 16 of LI-RADS v2018 CT/MRI Manual [27]
Nodule-in-nodule	See page 16–319 of Chapter 16 of LI-RADS v2018 CT/MRI Manual [27]
Mosaic architecture	See page 16–314 of Chapter 16 of LI-RADS v2018 CT/MRI Manual [27]
Fat in mass, more than adjacent liver	See page 16–323 of Chapter 16 of LI-RADS v2018 CT/MRI Manual [27]
Blood products in mass	See page 16–329 of Chapter 16 of LI-RADS v2018 CT/MRI Manual [27]
* Favoring benignity*	
Iron in mass, more than liver	See page 16–355 of Chapter 16 of LI-RADS v2018 CT/MRI Manual [27]
Marked T2 hyperintensity	See page 16–362 of Chapter 16 of LI-RADS v2018 CT/MRI Manual [27]
HBP isointensity	See page 16–369 of Chapter 16 of LI-RADS v2018 CT/MRI Manual [27]
Tumor in vein	See page 16–243 of Chapter 16 of LI-RADS v2018 CT/MRI Manual [27]
LR-M features	
* Targetoid appearances*	
Rim APHE	See page 16–38 of Chapter 16 of LI-RADS v2018 CT/MRI Manual [27]
Peripheral "washout"	See page 16–125 of Chapter 16 of LI-RADS v2018 CT/MRI Manual [27]
Delayed central enhancement	See page 16–221 of Chapter 16 of LI-RADS v2018 CT/MRI Manual [27]
Targetoid restriction	See page 16–234 of Chapter 16 of LI-RADS v2018 CT/MRI Manual [27]
Targetoid TP or HBP appearance	See page 16–227 of Chapter 16 of LI-RADS v2018 CT/MRI Manual [27]
* Nontargetoid features*	
Infiltrative appearance	See page 16–241 of Chapter 16 of LI-RADS v2018 CT/MRI Manual [27]
Marked diffusion restriction	See page 16–241 of Chapter 16 of LI-RADS v2018 CT/MRI Manual [27]
Necrosis or severe ischemia	See page 16–241 of Chapter 16 of LI-RADS v2018 CT/MRI Manual [27]
Other tumor-related prognostic features	
Intratumoral artery	Presence of discrete arterial enhancement within the tumor [28]
Incomplete tumor "capsule"	An absence of “capsule” or the presence of a disrupted “capsule” in any imaging plane [29]
Nonsmooth tumor margin	Non-nodular tumors or nodular tumors with irregular margin and budding portion at the tumor periphery in any imaging plane [28, 30]
Marked HBP hypointensity	Signal intensity of the liver observation in the HBP lower than that of liver and similar to or lower than that of vessels
HBP peritumoral hypointensity	Presence of wedge-shaped or flame-like hypointense area adjacent to the tumor border on HBP images [30]
HBP hypointense nodule without APHE	HBP hypointensity was defined as unequivocally darker signal intensity in HBP in whole or in part than liver, and APHE was defined as nonrim-like or rim enhancement in AP showing unequivocally higher intensity or attenuation in any part than liver [31]
Underlying liver disease-related features	
Radiological cirrhosis	Unequivocal morphological alterations of liver, including surface nodularity, small liver volume, expansion of space between liver and anterior abdominal wall and perihilar, gallbladder fossa and ligamentum teres spaces, hypertrophy of caudate and/or lateral left section, atrophy of anterior right section and/or medial left section, anterolateral flattening, notching of the posterior medial right lobe and parenchymal nodules, with or without manifestations of portal hypertension (portal-systemic collaterals, splenomegaly and/or ascites)
Diffuse fatty change	Diffuse signal intensity drop of the liver parenchyma on opposed-phase images compared with the in-phase images
Diffuse iron overload	Diffuse signal intensity drop of the liver parenchyma on in-phase images compared with the opposed-phase images
Width of main portal vein, cm	Diameter of the main portal vein, which is measured at least 1 cm distal to the confluence of splenic and superior mesenteric vein and at least 1 cm proximal to the first branch of the main portal vein, to avoid the effect of convergence/divergence, on coronal images [32]
Collateral circulation	Enhancing tortuous channels in esophageal, epigastric, perisplenic, paraumbilical, or retroperitoneal locations [33]
Gastroesophageal varices	Discrete enhancing tortuous channel abutting the luminal surface of the esophageal or gastric wall or contacting/protruding into luminal space [33]
Splenomegaly	Length > 13 cm [33]
Ascites	Presence of free fluid in the abdomen or pelvis [33]

*AP*, arterial phase; *APHE*, arterial phase hyperenhancement; *CT*, computed tomography; *HCC*, hepatocellular carcinoma; *HBP*, hepatobiliary phase; *LI-RADS/LR*, Liver Imaging Reporting and Data System; *MRI*, magnetic resonance imaging; *TP*, transitional phase

### Follow-up

OS was defined as the time interval from hepatectomy to death from any cause, and patients who were alive were censored at the date of the last follow-up. Follow-up ended on June 15, 2022, for institution 1 and July 30, 2021, for institution 2.

### Statistical analysis

Categorical variables were compared using the chi-square test or Fisher's exact test, while continuous variables were compared by the Student’s *t* test or Mann–Whitney *U* test, as appropriate. Interobserver agreement was assessed by computing Cohen’s κ statistics for binary features, weighted κ statistics for categorical features, and intraclass correlation coefficient for continuous variables, respectively.

#### Propensity score matching

Propensity score matching was performed to minimize the effects of potential confounders and selection bias between the training and internal validation cohorts [34]. The propensity score was estimated by logistic regression, with covariates including sex, age, liver cirrhosis, BCLC stage, and mortality as independent variables, and the type of study cohorts (training vs. internal validation cohorts) as the dependent variable for model fitting. Enrolled patients were matched using 1:1 optimal pair matching. The standardized mean difference was calculated to assess the covariate balance between the two matched cohorts, with a goal-to-achieve value  < 0.15. For the matched data, categorical variables were compared using the McNemar test, whilst continuous variables were compared using the paired *t* test or Wilcoxon signed-rank test, when applicable.

#### Development and validation of the risk score

Univariable Cox regression analysis was performed to identify potential predictors in the training cohort, whilst adjusting for patients’ sex and age. To develop an easy-to-apply score, continuous variables were transformed into binary variables as per normal ranges of laboratory indexes or clinical relevance. Spearman’s correlation coefficient was calculated to investigate the collinearity between variables; when collinearity was encountered, variables with the largest hazard ratios in the univariable analysis were selected for further analysis.

Variables with a *p* < 0.01 at univariable analysis were entered into the multivariable Cox regression model; the final model was selected using the backward stepwise approach with Akaike Information Criterion and five-fold cross-validation. A risk score was constructed based on the final multivariable Cox regression model. The effect of the variable with the highest β-coefficient was assigned 20 points, and all scaled β-coefficients were rounded to the nearest integer. Harrell’s concordance index (C-index) was used to measure the discrimination of the risk score [35], and the calibration curve was drawn to assess model calibration [36].

#### Survival analysis

OS was calculated by the Kaplan-Meier method and compared by the log-rank test, with a false discovery rate-adjusted approach applied [37]. To classify patients into high- and low-risk survival groups, the optimal cutoff value of the risk score was determined by X-tile software (version 3.6.1). Subgroup analyses were performed based on six available clinical-radiological-pathological variables known to affect HCC prognosis, including tumor size, tumor-in-vein, microvascular invasion (MVI), tumor differentiation, liver cirrhosis, and the ALBI grade. The prognostic value of the risk score was also assessed in subgroups of patients undergoing resection within (stage 0-A) and beyond (stage B-C) BCLC criteria. To ensure an adequate number of patients for each subgroup, the training and internal validation cohorts were used for subgroup analyses.

Statistical analyses were performed using R software (version 3.5.1; The R Foundation for Statistical Computing) and SPSS Statistics (version 26.0; IBM). A two-tailed *p* < 0.05 indicated a statistically significant difference.

## Results

### Patient characteristics

**Fig. 1:**
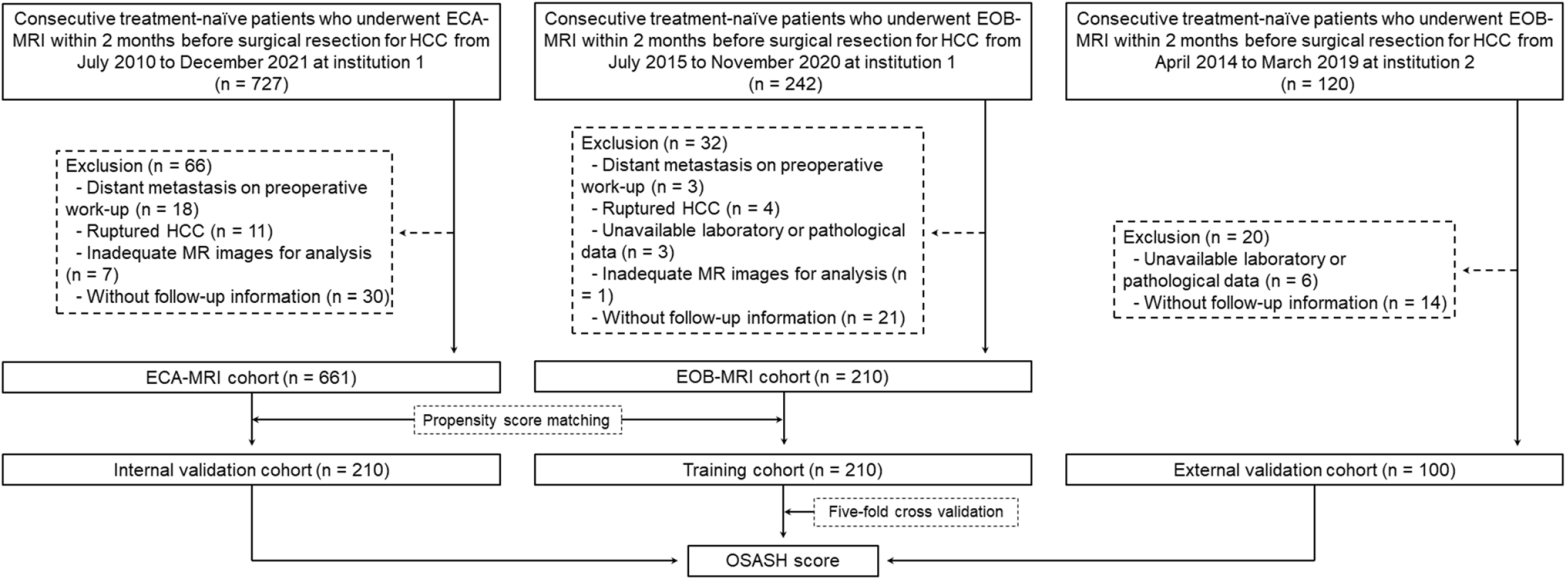
Flowchart of patient selection. EOB-MRI, gadoxetate disodium-enhanced magnetic resonance imaging; ECA-MRI, extracellular contrast agent-enhanced magnetic resonance imaging; HCC, hepatocellular carcinoma; MR, magnetic resonance

At institution 1, a total of 210 patients (mean age ± standard deviation, 52.1 ± 11.6 years; 173 men) in the “EOB-MRI cohort” constituted the training cohort. Given that no HBP imaging features were included in the final prognostic model, we matched 210 patients (mean age ± standard deviation, 53.5 ± 11.0 years; 178 men) in the “ECA-MRI cohort” for internal validation, with an adequate balance of all matching variables (Table 2 and S2). At institution 2, a total of 100 patients (mean age ± standard deviation, 56.2 ± 10.0 years; 81 men) were included and constituted the external validation cohort (Fig. 1).

**Table 2:** Baseline patient characteristics and MRI features

Characteristic	Training cohort (*n* = 210)	Internal validation cohort (*n* = 210)	*p* value^a^	External validation cohort (*n* = 100)	*p* value^b^
Age (y)^†^	52.1 ± 11.6	53.5 ± 11.0	0.180^#^	56.2 ± 10.0	0.002
Sex			0.598^#^		0.768
Female	37 (17.6)	32 (15.2)		19 (19.0)	
Male	173 (82.4)	178 (84.8)		81 (81.0)	
Cause of liver disease			0.040		0.208
HBV	194 (92.4)	181 (86.2)		88 (88.0)	
Others	16 (7.6)	29 (13.8)		12 (12.0)	
Cirrhosis	104 (49.5)	101 (48.1)	0.845^#^	70 (70.0)	0.001
Child–Pugh class			0.200		< 0.001
A	207 (98.6)	203 (96.7)		85 (85.0)	
B	3 (1.4)	7 (3.3)		15 (15.0)	
ALBI grade			0.103		< 0.001
1	170 (81.0)	155 (73.8)		30 (30.0)	
2	40 (19.0)	54 (25.7)		68 (68.0)	
3	0 (0.0)	1 (0.5)		2 (2.0)	
Laboratory index					
AST, IU/L^‡^	35.0 (27.0–48.0)	34.0 (26.8–50.0)	0.966	34.0 (26.2–50.7)	0.980
ALT, IU/L^‡^	37.0 (24.0–53.3)	35.5 (23.0–57.3)	0.899	41.0 (30.0–72.4)	0.002
TBIL, umol/L^‡^	13.5 (10.8–17.5)	13.4 (9.8–17.4)	0.282	14.9 (10.5–20.0)	0.116
ALB, g/L^‡^	42.9 (40.4–45.9)	42.7 (39.4–45.2)	0.230	37.6 (34.0–39.9)	< 0.001
PLT, × 10^9/L^‡^	137.5 (98.5–183.0)	126.5 (94.0–176.3)	0.182	124.0 (89.5–154.3)	0.034
PT, seconds^‡^	11.9 (11.1–12.6)	11.8 (11.2–12.5)	0.786	13.5 (12.5–14.9)	< 0.001
INR^‡,§^	1.0 (1.0–1.1)	1.0 (1.0–1.1)	0.438	NA (NA–NA)	…
ALP, IU/L^‡,§^	90.0 (72.0–115.0)	88.0 (69.0–115.3)	0.858	NA (NA–NA)	…
GGT, IU/L^‡,§^	49.5 (28.8–86.3)	52.5 (30.0–117.0)	0.103	NA (NA–NA)	…
AFP, ng/mL			0.601		0.001
≤ 400	145 (69.0)	140 (66.7)		87 (87.0)	
> 400	65 (31.0)	70 (33.3)		13 (13.0)	
BCLC stage			0.852^#^		0.702
0	29 (13.8)	28 (13.3)		13 (13.0)	
A	101 (48.1)	101 (48.1)		48 (48.0)	
B	34 (16.2)	40 (19.0)		21 (21.0)	
C	46 (21.9)	41 (19.5)		18 (18.0)	
MVI	96 (45.7)	90 (42.9)	0.556	53 (53.0)	0.230
Tumor differentiation			0.133		< 0.001
Well or Moderate	136 (64.8)	121 (57.6)		84 (84.0)	
Poor or undifferentiated	74 (35.2)	89 (42.4)		16 (16.0)	
Follow-up, months^‡^	48.1 (24.5–66.7)	51.3 (35.4–72.8)	< 0.001^*^	35.5 (33.3–49.2)	0.091^*^
No. of death	42 (20.0)	41 (19.5)	1.000^#^	20 (20.0)	1.000
MRI features					
Tumor burden-related features					
Tumor multiplicity			0.080		0.557
1	134 (63.8)	147 (70.0)		58 (58.0)	
2 or 3	43 (20.5)	45 (21.4)		22 (22.0)	
≥ 4	33 (15.7)	18 (8.6)		20 (20.0)	
Tumor size, cm^‡^	4.1 (2.4–6.9)	4.2 (2.8–7.1)	0.411	3.4 (2.2–4.9)	0.022
Bilobar involvement	29 (13.8)	20 (9.5)	0.171	28 (28.0)	0.003
LI-RADS major features					
Nonrim APHE	199 (94.8)	194 (92.4)	0.320	85 (85.0)	0.004
Nonperipheral "washout"	194 (92.4)	148 (70.5)	< 0.001	91 (91.0)	0.676
Enhancing "capsule"	139 (66.2)	170 (81.0)	0.001	55 (55.0)	0.057
LI-RADS ancillary features					
* Favoring malignancy in general, not HCC in particular*					
Corona enhancement	81 (38.6)	102 (48.6)	0.039	38 (38.0)	0.923
Fat sparing in solid mass^§§§^	9 (4.3)	8 (3.8)	0.804	NA (NA)	…
Diffusion restriction	210 (100.0)	210 (100.0)	…	100 (100.0)	…
Mild-moderate T2 hyperintensity	206 (98.1)	209 (99.5)	0.372	93 (93.0)	0.053
Iron sparing in solid mass	26 (12.4)	29 (13.8)	0.664	33 (33.0)	< 0.001
TP hypointensity^§§^	205 (97.6)	NA (NA)	…	95 (95.0)	0.381
HBP hypointensity^§§^	204 (97.1)	NA (NA)	…	98 (98.0)	0.951
* Favoring HCC in particular*					
Nonenhancing "capsule"	40 (19.0)	1 (0.5)	< 0.001	1 (1.0)	< 0.001
Nodule-in-nodule	74 (35.2)	87 (41.4)	0.192	18 (18.0)	0.002
Mosaic architecture	92 (43.8)	70 (33.3)	0.027	29 (29.0)	0.012
Fat in mass, more than adjacent liver^§§§^	82 (39.0)	79 (37.6)	0.763	NA (NA)	…
Blood products in mass	79 (37.6)	81 (38.6)	0.841	30 (30.0)	0.189
* Favoring benignity*					
Iron in mass, more than liver	3 (1.4)	0 (0.0)	0.248	4 (4.0)	0.218
Marked T2 hyperintensity	3 (1.4)	3 (1.4)	1.000	5 (5.0)	0.141
HBP isointensity^§§^	4 (1.9)	NA (NA)	…	2 (2.0)	1.000
Tumor in vein	44 (21.0)	41 (19.5)	0.716	15 (15.0)	0.212
LR-M features					
* Targetoid appearances*					
Rim APHE	10 (4.8)	11 (5.2)	0.823	12 (12.0)	0.020
Peripheral "washout"	1 (0.5)	0 (0.0)	1.000	2 (2.0)	0.244
Delayed central enhancement	2 (1.0)	11 (5.2)	0.011	6 (6.0)	0.025
Targetoid restriction	0 (0.0)	8 (3.8)	0.007	3 (3.0)	0.033
Targetoid TP or HBP appearance^§§^	2 (1.0)	NA (NA)	…	0 (0.0)	1.000
* Nontargetoid features*					
Infiltrative appearance	42 (20.0)	42 (20.0)	1.000	22 (22.0)	0.684
Marked diffusion restriction	94 (44.8)	34 (16.2)	< 0.001	53 (53.0)	0.174
Necrosis or severe ischemia	72 (34.3)	95 (45.2)	0.022	36 (36.0)	0.767
LIRADS category			0.071		0.002
LR-3	1 (0.5)	0 (0.0)		2 (2.0)	
LR-4	12 (5.7)	22 (10.5)		3 (3.0)	
LR-5	185 (88.1)	169 (80.5)		77 (77.0)	
LR-M	12 (5.7)	19 (9.0)		18 (18.0)	
Other tumor-related prognostic features					
Intratumoral artery	71 (33.8)	77 (36.7)	0.540	25 (25.0)	0.117
Incomplete tumor "capsule"	138 (65.7)	164 (78.1)	0.005	79 (79.0)	0.017
Nonsmooth tumor margin	131 (62.4)	166 (79.0)	< 0.001	95 (95.0)	< 0.001
Marked HBP hypointensity^§§^	172 (81.9)	NA (NA)	…	92 (92.0)	0.019
HBP peritumoral hypointensity^§§^	79 (37.6)	NA (NA)	…	20 (20.0)	0.002
HBP hypointense nodule without APHE^§§^	73 (34.8)	NA (NA)	…	25 (25.0)	0.084
Underlying liver disease-related features					
Radiological cirrhosis	94 (44.8)	128 (61.0)	0.001	63 (63.0)	0.003
Diffuse fatty change^§§§^	19 (9.0)	13 (6.2)	0.270	NA (NA)	< 0.001
Diffuse iron overload	52 (24.8)	39 (18.6)	0.124	35 (35.0)	0.061
Width of main portal vein, cm^‡^	1.5 (1.3–1.6)	1.5 (1.3–1.6)	0.207	1.5 (1.3–1.6)	0.570
Collateral circulation	112 (53.3)	127 (60.5)	0.139	59 (59.0)	0.348
Gastroesophageal varices	52 (24.8)	113 (53.8)	< 0.001	59 (59.0)	< 0.001
Splenomegaly	85 (40.5)	94 (44.8)	0.375	43 (43.0)	0.673
Ascites	0 (0.0)	20 (9.5)	< 0.001	9 (9.0)	< 0.001

Unless indicated otherwise, data are the number of patients, with percentages in parentheses. Group comparisons were performed with the Student’s *t* test or Mann–Whitney *U* test for continuous variables and chi-square or Fisher's exact test for categorical variables, as appropriate

^#^Group comparisons were performed with the paired t test or Wilcoxon signed rank test for continuous variables and McNemar test for categorical variables, as appropriate. *Group comparisons were performed with the log-rank test

^a^Training vs. internal validation cohorts. ^b^Training vs. external validation cohorts

^†^Data are means ± standard deviations. ‡Data are medians, with interquartile range in parentheses. §Data were unavailable in the external validation cohort. §§Data were unavailable in the internal validation cohort owing to the lack of TP and HBP images on ECA-MRI. §§§Data were unavailable in the external validation cohort owing to the lack of T1-weighted in- and opposed-phase images

*AFP*, alpha-fetoprotein; *ALB*, albumin; *ALBI*, albumin-bilirubin; *ALP*, alkaline phosphatase; *ALT*, alanine aminotransferase; *APHE*, arterial phase hyperenhancement; *AST*, aspartate aminotransferase; *BCLC*, Barcelona Clinic Liver Cancer; *GGT*, gamma-glutamyl transferase; *HBP*, hepatobiliary phase; *HBV*, hepatitis B virus; *HCC*, hepatocellular carcinoma; *INR*, international normalized ratio; *LI-RADS/LR*, Liver Imaging Reporting and Data System; *MRI*, magnetic resonance imaging; *MVI*, microvascular invasion; *NA*, not available; *PLT*, platelet; *PT*, prothrombin time; *TBIL*, total bilirubin; *TP*, transitional phase

Patients from the training cohort were younger than those from the external validation cohort (mean age, 52.1 vs. 56.2 years; *p* = 0.002), with less frequent cirrhosis (49.5 vs. 70.0%; *p* = 0.001) and larger tumors (median size, 4.1 vs. 3.4 cm; *p* = 0.022). The median follow-up period was 48.1 (interquartile range [IQR]: 24.5–66.7), 51.3 (IQR: 35.4–72.8), and 35.5 (IQR: 33.3–49.2) months for the training, internal validation and external validation cohorts, respectively. Baseline patient characteristics and MRI features are summarized in Table 2.

### Development and validation of the OSASH score for predicting OS

**Fig. 2:**
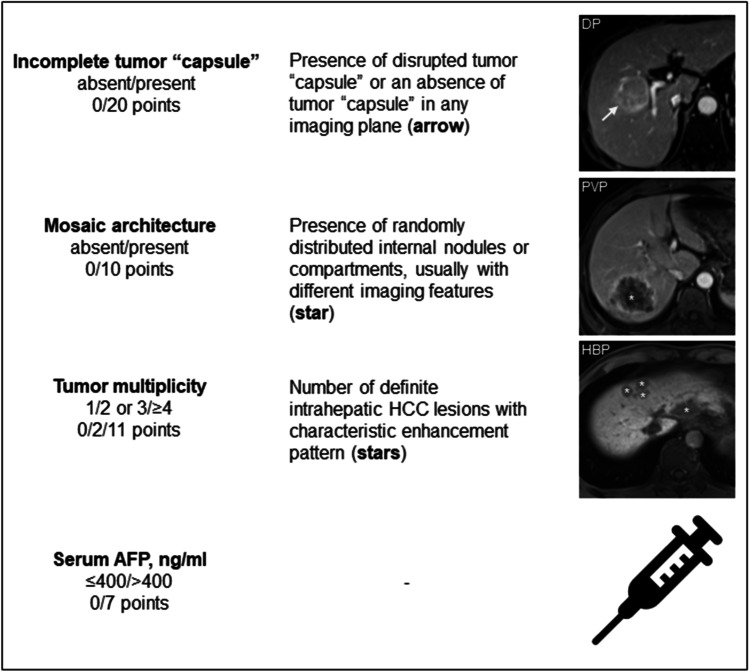
Graphical illustration of the OSASH score. AFP, alpha-fetoprotein; DP, delayed phase; HBP, hepatobiliary phase; HCC, hepatocellular carcinoma; PVP, portal venous phase

In the training cohort, 11 variables were significantly associated with worse OS at univariable Cox regression analysis, and four of them (i.e., incomplete tumor “capsule”, mosaic architecture, tumor multiplicity, and serum AFP  > 400 ng/mL) were included in the final model at multivariable analysis (Table 3). By incorporating these variables, a simplified risk score termed the “OSASH score” (named by incorporating the key letters of *o*verall *s*urvival *a*fter *s*urgery for *HCC*) was developed (Fig. 2).

**Table 3:** Univariable and multivariable Cox regression analysis of prognostic factors for overall survival in the training cohort

	Univariable analysis		Multivariable analysis			
Variable	Hazard ratio (95%CI)	*p* value	Hazard ratio (95%CI)	β-estimate (95%CI)	*p* value	Point
Age	0.97 (0.94–0.99)	0.018				
Sex, female vs. male	0.74 (0.36–1.50)	0.403				
Etiology, HBV vs. non-HBV	1.06 (0.33–3.43)	0.923				
Cirrhosis, absent vs. present	0.73 (0.40–1.35)	0.322				
ALBI grade, 1 vs. 2	0.65 (0.27–1.55)	0.331				
AST, ≤ 40 vs. > 40 IU/L	1.95 (1.06–3.57)	0.031				
ALT, ≤ 50 vs. > 50 IU/L	1.29 (0.67–2.49)	0.441				
TBIL, ≤ 19 vs. > 19 umol/L	0.65 (0.27–1.55)	0.333				
ALB, ≥ 40 vs. < 40 g/L	0.73 (0.34–1.57)	0.414				
PLT, ≥ 100 vs. < 100 × 10^9/L	0.61 (0.27–1.37)	0.23				
PT, ≤ 13 vs. > 13 s	1.33 (0.61–2.88)	0.471				
INR, ≤ 1.1 vs. > 1.1	0.94 (0.43–2.03)	0.87				
ALP, ≤ 160 vs. > 160 IU/L	1.48 (0.45–4.81)	0.518				
GGT, ≤ 60 vs. > 60 IU/L	3.10 (1.67–5.75)	< 0.001				
AFP, ≤ 400 vs. > 400 ng/mL	4.10 (2.21–7.60)	< 0.001	2.26 (1.18–4.31)	0.81 (0.17–1.46)	0.014	7
Tumor multiplicity						
1	Reference		Reference			
2 or 3	3.24 (1.40–7.47)	0.006	1.26 (0.53–3.04)	0.23 (–0.64–1.11)	0.600	2
≥ 4	10.11 (4.83–21.13)	< 0.001	3.36 (1.51–7.45)	1.21 (0.41–2.01)	0.003	11
Tumor size^†^	1.22 (1.14–1.31)	< 0.001				
Enhancing "capsule", absent vs. present	0.72 (0.39–1.33)	0.297				
Corona enhancement, absent vs. present	3.67 (1.93–6.97)	< 0.001				
Iron sparing in solid mass, absent vs. present	0.95 (0.37–2.43)	0.919				
Mosaic architecture, absent vs. present^†^	6.55 (2.91–14.76)	< 0.001	2.99 (1.28–6.95)	1.09 (0.25–1.94)	0.011	10
Fat in mass, more than adjacent liver, absent vs. present	0.47 (0.24–0.94)	0.033				
Blood products in mass, absent vs. present	4.16 (2.16–8.00)	< 0.001				
Tumor in vein, absent vs. present	4.69 (2.56–8.60)	< 0.001				
Infiltrative appearance, absent vs. present	5.69 (3.08–10.53)	< 0.001				
Marked diffusion restriction, absent vs. present	2.05 (1.10–3.82)	0.024				
Necrosis or severe ischemia, absent vs. present	1.87 (1.02–3.43)	0.042				
LIRADS category, LR-3/4/5 vs. LR-M	2.45 (0.96–6.25)	0.060				
Bilobar involvement, absent vs. present	2.23 (1.12–4.43)	0.023				
Intratumoral artery, absent vs. present	4.15 (2.20–7.80)	< 0.001				
Incomplete tumor "capsule", absent vs. present^§^	25.36 (3.49–184.44)	0.001	8.99 (1.16–69.32)	2.20 (0.15–4.24)	0.035	20
Nonsmooth tumor margin, absent vs. present^§^	13.84 (3.34–57.32)	< 0.001				
Marked HBP hypointensity, absent vs. present	3.11 (0.96–10.08)	0.058				
HBP peritumoral hypointensity, absent vs. present	3.50 (1.84–6.66)	< 0.001				
HBP hypointense nodule without APHE	1.12 (0.60–2.09)	0.725				
Radiological cirrhosis, absent vs. present	0.76 (0.41–1.41)	0.379				
Diffuse iron overload, absent vs. present	0.49 (0.19–1.24)	0.131				
Width of main portal vein, cm	0.34 (0.08–1.48)	0.149				
Collateral circulation, absent vs. present	1.33 (0.72–2.46)	0.368				
Gastroesophageal varices, absent vs. present	1.34 (0.70–2.58)	0.383				
Splenomegaly, absent vs. present	0.94 (0.50–1.75)	0.850				

The OSASH score for an individual patient could be calculated by the following formula:

**OSASH score** = **Incomplete tumor “capsule”** (absent = 0; present = 20) + **Mosaic architecture** (absent = 0; present = 10) + **Tumor multiplicity** (solitary = 0; two or three = 2; four or more = 11) + **AFP** (≤ 400 ng/mL = 0; > 400 ng/mL = 7)

^†^Due to significant collinearity (*r*_*s*_ = 0.715, *p* < 0.001), “mosaic architecture” was entered into the multivariable Cox regression model owing to the largest hazard ratio. §Due to significant collinearity (*r*_*s*_ = 0.744, *p* < 0.001), "incomplete tumor “capsule” was entered into the multivariable Cox regression model owing to the largest hazard ratio

*AFP*, alpha-fetoprotein; *ALB*, albumin; *ALBI*, albumin-bilirubin; *ALP*, alkaline phosphatase; *ALT*, alanine aminotransferase; *APHE*, arterial phase hyperenhancement; *AST*, aspartate aminotransferase; *CI*, confidence interval; *GGT*, gamma-glutamyl transferase; *HBP*, hepatobiliary phase; *HBV*, hepatitis B virus; *INR*, international normalized ratio; *LI-RADS/LR*, Liver Imaging Reporting and Data System; *MVI*, microvascular invasion; *PLT*, platelet; *PT*, prothrombin time; *TBIL*, total bilirubin

The C-index of the OSASH score for predicting OS was 0.85 (95% confidence interval [CI]: 0.78–0.91), 0.81 (95% CI: 0.75–0.88), and 0.62 (95% CI: 0.49–0.75) in the training, internal validation and external validation cohorts, respectively. The calibration plots showed an overall good agreement between the score-predicted risk of death and the observed incidence of death in all cohorts (Figure S1). Interobserver agreement for the OSASH score was presented in Table S3.

### Survival risk stratification based on the OSASH score

**Fig. 3:**
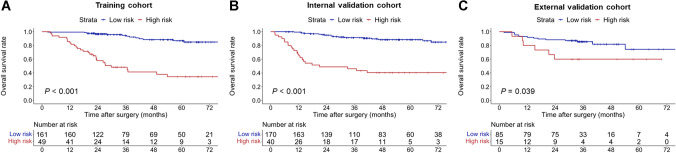
Kaplan-Meier curves demonstrating differences in OS between the OSASH-low and OSASH-high risk patients with HCC in the (**A**) training, (**B**) internal validation, and (**C**) external validation cohorts. HCC, hepatocellular carcinoma; OS, overall survival

Using 32 as the cutoff point for the OSASH score derived from the training cohort (Table 4; Fig. 3A), patients in the internal validation cohort were divided into prognostically distinct low and high-risk groups (5-year OS rates, 88.5% vs. 40.5%; *p* < 0.001) (Table 4; Fig. 3B). Similar results were also obtained in the external validation cohort (5-year OS rates, 74.3% vs. 60.0%; *p* = 0.039) (Table 4; Fig. 3C).

**Table 4:** Median OS, 3- and 5-year OS rates, and hazard ratios for OSASH score risk subclasses in all cohorts

Cohort and risk group	No	Median OS, months (95%CI)	3-year OS rate, % (95%CI)	5-year OS rate, % (95%CI)	Hazard Ratio (95%CI)	*p* value
Training cohort						< 0.001
Low risk	161	NA (NA–NA)	94.6 (90.6–98.7)	86.9 (80.1–94.2)	Reference	
High risk	49	29.7 (23.3–NA)	48.3 (35.7–65.4)	34.5 (22.0–54.0)	9.16 (4.81–17.45)	
Internal validation cohort						< 0.001
Low risk	170	NA (NA–NA)	91.4 (87.0–96.0)	88.5 (83.2–94.1)	Reference	
High risk	40	21.0 (12.6–NA)	46.1 (32.7–64.9)	40.5 (27.5–59.6)	8.36 (4.49–15.56)	
External validation cohort						0.039
Low risk	85	NA (NA–NA)	85.5 (78.2–93.5)	74.3 (59.4–93.1)	Reference	
High risk	15	NA (21.3–NA)	60.0 (39.7–90.7)	60.0 (39.7–90.7)	2.64 (1.01–6.90)	

*Captions:*

*CI*, confidence interval; *NA*, not available; *OS*, overall survival

### The OSASH score predicted OS in six subgroups

In each subgroup, as mentioned above, OSASH-low-risk patients had significantly longer OS than that of OSASH-high risk patients in both the training (Table 5; Figure S2) and internal validation (Table 5; Figure S3) cohorts (all *p* < 0.05).

**Table 5:** 5-year OS rates and hazard ratios for OSASH score risk subclasses in six subgroups in the training and internal validation cohorts

Variable and risk group	Training cohort (*n* = 210)				Internal validation cohort (*n* = 210)			
	No	5-year OS rate, % (95%CI)	Hazard ratio (95%CI)	*p* value	No	5-year OS rate, % (95%CI)	Hazard Ratio (95%CI)	*p* value
Tumor diameter, ≤ 5 cm				< 0.001				< 0.001
Low risk	114	88.4 (80.0–97.7)	Reference		119	93.5 (89.0–98.3)	Reference	
High risk	9	44.4 (17.6–100.0)	8.94 (2.60–30.65)		5	40.0 (13.7–100.0)	12.98 (3.35–50.31)	
Tumor diameter, > 5 cm				< 0.001				< 0.001
Low risk	47	83.7 (72.4–96.8)	Reference		51	74.5 (60.9–91.2)	Reference	
High risk	40	33.0 (19.9–54.6)	6.16 (2.64–14.38)		35	40.9 (27.2–61.6)	4.13 (1.92–8.86)	
Tumor-in-vein, absent				< 0.001				0.013
Low risk	147	89.5 (83.1–96.5)	Reference		151	93.4 (89.0–98.0)	Reference	
High risk	19	34.6 (17.5–68.6)	9.54 (4.05–22.49)		18	76.2 (58.2–99.7)	3.93 (1.23–12.62)	
Tumor-in-vein, present				0.040				< 0.001
Low risk	14	67.5 (45.4–100.0)	Reference		19	52.5 (32.8–84.3)	Reference	
High risk	30	38.3 (23.5–62.5)	2.99 (1.00–8.96)		22	13.6 (4.8–39.0)	4.16 (1.80–9.62)	
MVI, absent				0.002				< 0.001
Low risk	108	92.7 (86.6–99.3)	Reference		113	95.0 (90.7–99.4)	Reference	
High risk	6	62.5 (32.0–100.0)	8.86 (1.71–45.96)		7	57.1 (30.1–100.0)	9.95 (2.47–40.12)	
MVI, present				< 0.001				< 0.001
Low risk	53	74.3 (59.4–92.9)	Reference		57	73.1 (59.9–89.3)	Reference	
High risk	43	31.0 (18.4–52.2)	4.84 (2.26–10.34)		33	36.8 (23.1–58.6)	4.18 (2.03–8.62)	
Well or moderate tumor differentiation				< 0.001				< 0.001
Low risk	116	91.5 (84.9–98.6)	Reference		108	89.8 (83.6–96.5)	Reference	
High risk	20	36.3 (17.5–75.1)	11.55 (4.18–31.87)		13	44.0 (23.3–83.0)	8.75 (3.37–22.72)	
Poor tumor differentiation				< 0.001				< 0.001
Low risk	45	76.8 (62.4–94.5)	Reference		62	86.3 (77.3–96.5)	Reference	
High risk	29	32.5 (18.3–57.9)	5.39 (2.33–12.48)		27	39.1 (24.1–63.4)	7.42 (3.04–18.10)	
Cirrhosis, absent				< 0.001				< 0.001
Low risk	75	83.5 (73.2–95.2)	Reference		94	89.0 (82.0–96.7)	Reference	
High risk	31	36.6 (20.2–66.2)	6.83 (2.90–16.09)		15	59.3 (38.7–90.7)	5.31 (1.88–14.97)	
Cirrhosis, present				< 0.001				< 0.001
Low risk	86	89.8 (81.2–99.3)	Reference		76	88.0 (80.6–96.2)	Reference	
High risk	18	30.9 15.1–63.1)	12.33 (4.62–32.96)		25	29.5 (15.7–55.5)	10.01 (4.30–23.30)	
ALBI grade, 1				< 0.001				< 0.001
Low risk	130	85.5 (77.4–94.4)	Reference		128	89.8 (84.2–95.9)	Reference	
High risk	40	32.2 (19.1–54.0)	8.79 (4.39–17.62)		27	51.2 (35.2–74.3)	7.46 (3.34–16.69)	
ALBI grade, 2 or 3				0.001				< 0.001
Low risk	31	91.4 (80.3–100.0)	Reference		42	84.6 (72.9–98.2)	Reference	
High risk	9	46.7 (21.0–100.0)	10.38 (1.86–57.76)		13	17.3 (4.9–60.6)	12.77 (4.27–38.20)	

*ALBI*, albumin-bilirubin; *CI*, confidence interval; *HCC*, hepatocellular carcinoma; *MVI*, microvascular invasion; *NA*, not available; *OS*, overall survival

### Prognostic impact of the OSASH score in patients across different BCLC stages

#### Survival risk stratification based on the BCLC algorithm

**Fig. 4:**
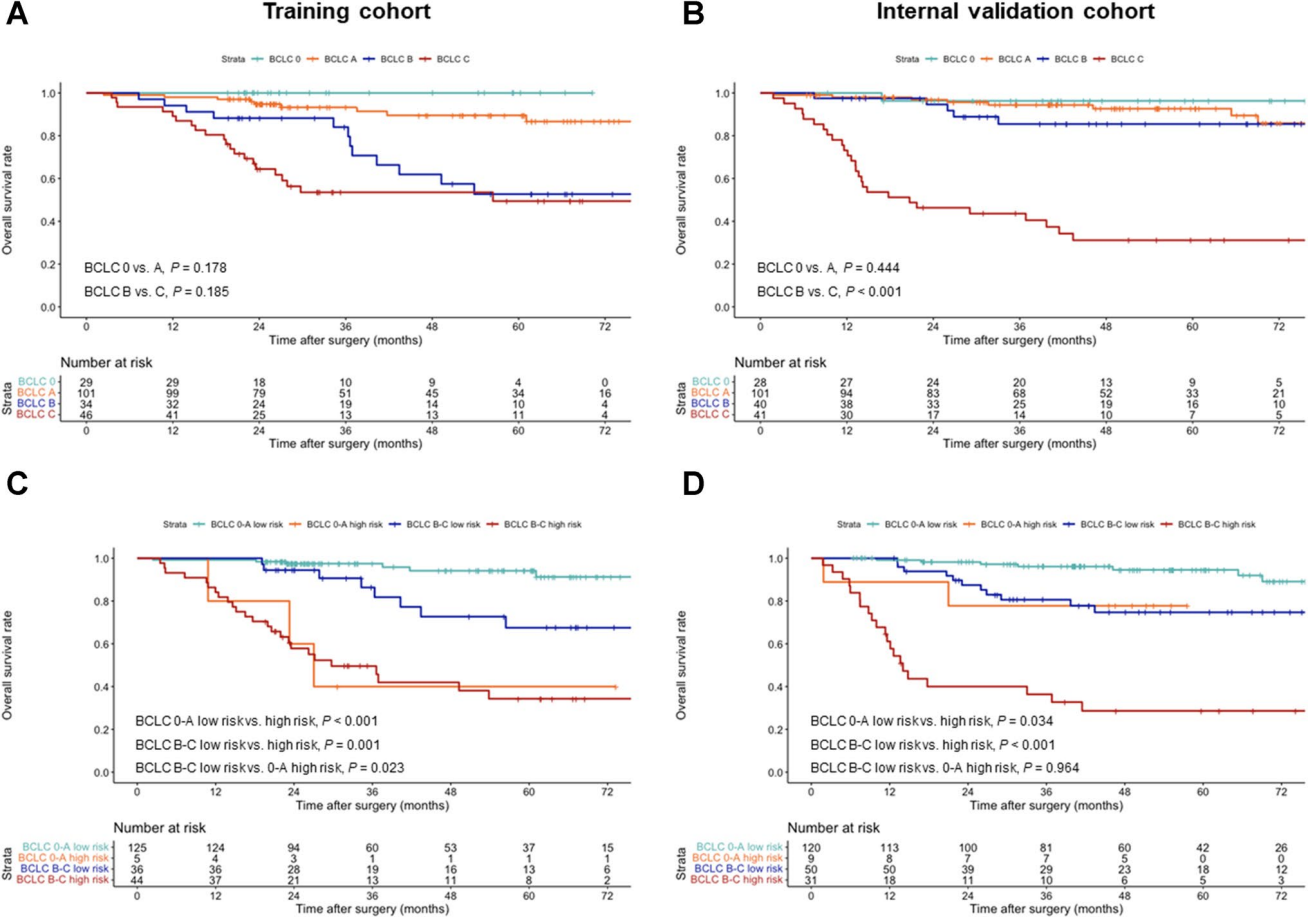
Kaplan-Meier curves according to (**A**, **B**) BCLC stage and (**C**, **D**) the OSASH score combined with BCLC stage subgroups in the training and internal validation cohorts. BCLC, Barcelona Clinic Liver Cancer

In the training cohort, comparable outcomes were obtained for patients with BCLC stage 0 and A HCC (5-year OS rates, 100.0% vs. 89.6%; *p* = 0.178) and for patients with BCLC stage B and C HCC (5-year OS rates, 52.7% vs. 49.4%; *p* = 0.185) (Table S4; Fig. 4A). In the internal validation cohort, patients with BCLC stage 0 and A HCC had similar outcomes (5-year OS rates, 96.3% vs. 92.7%; *p* = 0.444), whereas patients with BCLC stage B HCC had significantly longer OS than that of those with BCLC stage C HCC (5-year OS rates, 85.5% vs. 31.2%; *p* < 0.001) (Table S4; Fig. 4B).

#### Incremental prognostic value of the OSASH score to the BCLC algorithm

To further assess the incremental value of the OSASH score to the BCLC algorithm, subgroup analyses were performed in patients with BCLC stage 0-A and B-C HCC. In both the training and internal validation cohorts, OSASH-low-risk patients achieved significantly longer OS than that of OSASH-high-risk patients in either patients with BCLC stage 0-A HCC (internal validation cohort: 5-year OS rates, 94.6% vs. 77.8%; *p* = 0.034) or with BCLC stage B-C HCC (internal validation cohort: 5-year OS rates, 74.7% vs. 28.7%; *p* < 0.001) (Table S4; Fig. 4C and D).

Furthermore, in the training cohort, patients with BCLC stage B-C HCC and OSASH-low risk had significantly longer OS than those with BCLC stage 0-A HCC and OSASH-high risk (5-year OS rates, 67.5% vs. 40.0%; *p* = 0.023) (Fig. 4C). In the internal validation cohort, patients with BCLC stage B-C HCC and OSASH-low risk achieved comparable outcomes to that of those with BCLC stage 0-A HCC and OSASH-high risk (5-year OS rates, 74.7% vs. 77.8%; *p* = 0.964) (Fig. 4D and S4).

## Discussion

In this large dual-institutional cohort study, by integrating three MRI features and serum AFP, we developed and validated a preoperative risk score for the prediction of OS in HCC patients undergoing hepatectomy. The score displayed similarly good prognostic performance in the training and internal validation cohorts but a reduced performance in the external validation cohort. It was capable of stratifying patients into 2 prognostically distinct risk strata among all study cohorts and six subgroups. This was clinically relevant because it may allow the identification of a small portion of patients at high risk of death, for whom more intensive surveillance could be considered, and adjuvant therapies might provide survival benefit. Furthermore, among patients with BCLC stage B and C HCC without extrahepatic metastasis, this score identified a subgroup of low-risk patients who achieved favorable prognoses after resection, suggesting its potential value to complement traditional staging systems for OS prediction.

In the present study, 38.5% (200/520) of surgical patients had intermediate- to advanced-stage HCC. Despite representing a marked deviation from the current BCLC recommendations [3], our study population captured the context of real-world clinical practice of high-volume tertiary care centers in China, where a proportion of patients underwent hepatectomy for intermediate- to advanced-stage HCC according to the multidisciplinary team recommendations and patients’ preferences [7]. However, patients with intermediate- to advanced-stage HCC harbor substantial tumor heterogeneity, hence a preoperative patient selection is critical to identify the optimal surgical candidates. In our study, patients with BCLC stage B-C HCC and OSASH-low risk might be potential surgical candidates because these patients showed 5-year OS rates approaching 70%, which were similar to patients with BCLC 0-A HCC and OSASH-high risk. However, future prospective multi-institutional studies are required to test the reliability and reproducibility of our findings.

To date, EOB-MRI has been more commonly used in published prognostic researches, mainly because it can additionally provide HBP imaging features for analysis, of which some (e.g., HBP peritumoral hypointensity) have been closely linked to HCC prognosis [20, 23, 38]. Therefore, in the current study, to comprehensively explore the prognostic value of all available imaging features, the risk score was initially developed in an EOB-MRI cohort (the training cohort). However, our results showed that no HBP imaging features were independently associated with OS, which motivated us to test the reproducibility of our findings in a propensity score-matched ECA-MRI cohort. It turned out that the ECA-MRI cohort showed comparably discriminatory power as the EOB-MRI cohort. These preliminary observations suggest that the risk score was applicable for both EOB-MRI and ECA-MRI to help predict OS for HCC patients undergoing surgical resection.

The OSASH score was constructed with 4 variables profiling the tumor burden (tumor multiplicity) and biology (incomplete tumor “capsule,” mosaic architecture, and AFP). The mechanisms underlying these clinic-radiological alterations are still in research. The presence of an incomplete tumor “capsule” often indicates infiltrative tumor growth and poorer survival [39]. Previous studies have identified incomplete tumor “capsule” as an imaging marker for predicting MVI [40], postoperative extrahepatic metastasis [41], and high *BRAF* and *RAF1* expression in HCC [42], and the latter could accelerate tumor proliferation and differentiation and promote tumor invasion and metastasis. Mosaic architecture refers to the presence of randomly distributed internal nodules or components, usually with different imaging features in terms of enhancement, intensity, shape, and size [27]. Histopathologically, it corresponds to the appearance of different foci of clonal expansion at various stages of hepatocarcinogenesis, of which some may comprise fat metamorphosis, necrosis, blood products, cystic degeneration, and fibrosis septa [43]. Therefore, mosaic architecture is regarded as an imaging marker of tumor heterogeneity at the histological level, while the latter is a critical prognostic element. Serum AFP  > 400 ng/mL also denoted a worse OS in our study, keeping in line with previous reports [44, 45]. AFP can promote tumor growth partly by the inhibition of apoptosis; besides, it was also associated with the upregulation of vascular endothelial growth factor signaling, thereby promoting tumor angiogenesis and metastasis [46].

This study had several limitations. Firstly, there might have been unavoidable selection bias owing to the retrospective design. Particularly, our patients were enrolled over a time span of 11 years, which could be a potential source of bias given the evolutions in MRI and surgical techniques. Apart from that, there was a time difference of 1 year in the last follow-up time between the two institutions, which might have impacted our results. Thus, further prospective multicenter studies are warranted to validate the presented findings. Secondly, the OSASH score did not achieve a good prognostic performance in the external validation cohort, which might have been due to the relatively small external sample size and the substantial heterogeneities in the study population, MRI parameters, and surgical techniques between the two institutions. Therefore, future large-scale studies are needed to verify the generalizability of the OSASH score in different populations. Thirdly, due to the smaller sample size in the external validation cohort, it was impossible to conduct further survival analyses to examine whether OSASH-low-risk patients had better outcomes than OSASH-high-risk patients in different subgroups, as shown in the internal validation cohort. Hence, the predictive ability of the OSASH score in various subgroups requires to be further externally validated using another larger cohort.

In conclusion, by incorporating three preoperative MRI features and serum AFP, we developed and validated the OSASH score for the prediction of postsurgical OS in HCC patients, which identified a subgroup of low-risk patients with BCLC stage B and C HCC who achieved favorable prognosis after resection. Future multicenter prospective studies with rigorous design are needed to validate our findings.

### Supplementary Information

Below is the link to the electronic supplementary material.

Supplementary file1 (PDF 2343 KB)

## Abbreviations

AFP: Alpha-fetoprotein
ALBI: Albumin-bilirubin
BCLC: Barcelona Clinic Liver Cancer
C-index: Concordance index
CI: Confidence interval
ECA-MRI: Extracellular contrast agent-enhanced magnetic resonance imaging
EOB-MRI: Gadoxetate disodium-enhanced magnetic resonance imaging
HBP: Hepatobiliary phase
HCC: Hepatocellular carcinoma
INR: International normalized ratio
IQR: Interquartile range
LI-RADS: Liver Imaging Reporting and Data System
MR: Magnetic resonance
MRI: Magnetic resonance imaging
MVI: Microvascular invasion
OS: Overall survival
PT: Prothrombin time

## Declarations

### Guarantor

The scientific guarantor of this publication is Bin Song, M.D.

### Statistics and biometry

One co-author (Yuanan Wu, Big Data Research Center, University of Electronic Science and Technology of China, Chengdu, Sichuan, China) has significant statistical expertise.

### Study subjects or cohorts overlap

Some study subjects (35%, 184/520) have been previously reported in another study. The prior work proposed an MRI-based risk score for prediction of HCC recurrence after hepatectomy, while the current work aimed to develop a risk score based on preoperative clinical-radiological features for prediction of overall survival and to investigate its utility in identifying patients with intermediate- to advanced-stage HCC who would potentially benefit from liver resection.

### Methodology

• retrospective

• diagnostic or prognostic study

• multicenter study
