# Supplementary material for: Development and validation of the OSASH score to predict overall survival of hepatocellular carcinoma after surgical resection: a dual-institutional study
Source: Eur Radiol. 2023 May 16;33(11):7631–45. doi: 10.1007/s00330-023-09725-7 (PMC10598081; doi:10.1007/s00330-023-09725-7)
Supplement: Supplementary file 1 — Supplementary file1 (PDF 2343 KB) [file 330_2023_9725_MOESM1_ESM.pdf]

ELECTRONIC SUPPLEMENTARY MATERIAL

Development and validation of the OSASH score to predict overall survival of hepatocellular carcinoma after surgical resection: A dual-institutional study

Table of Contents

Supplementary Material 1 MRI technique 1

Table S1 MRI sequences and parameters 2

Table S2 Characteristics of study patients before PSM and SMDs before and after PSM 5

Table S3 Interobserver agreement for the OSASH score 6

Table S4 Median OS, 3- and 5-year OS rates, and hazard ratios for BCLC stage subgroups and OSASH score risk subclasses in patients with BCLC stage 0-A and B-C HCC in the training and internal validation cohorts 7

Figure S1 Calibration plots for the OSASH score in predicting 3-year overall survival in all cohorts 8

Figure S2 Kaplan-Meier curves demonstrating differences in OS between the OSASH-low and OSASH-high risk patients with HCC in six subgroups in the training cohort 9

Figure S3 Kaplan-Meier curves demonstrating differences in OS between the OSASH-low and OSASH-high risk patients with HCC in six subgroups in the internal validation cohort 10

Figure S4 A OSASH-low risk patient with BCLC stage C HCC in the internal validation cohort 11

Supplementary Material 1

MRI technique

At institution 1, gadoxetate disodium-enhanced magnetic resonance imaging (EOB-MRI) was performed with four 3.0-T systems (GE SIGNA™ Architect; GE SIGNA™ Premier; GE Discovery MR 750; Siemens MAGNETOM Skyra) and a 1.5-T system (uMR588), and extracellular contrast agent-enhanced MRI was performed with five 3.0-T systems (Siemens MAGNETOM Skyra; Siemens TrioTim; GE SIGNA™ Architect; GE Discovery MR 750; Philips Ingenia Elition X) and two 1.5-T systems (Siemens Avanto; uMR588). At institution 2, EOB-MRI was performed with a 3.0-T system (GE Discovery MR 750). Liver MRI protocols involved T2-weighted imaging, diffusion-weighted imaging (b values: 0-1200 s/mm^2 at institution 1; 0-3000 s/mm^2 at institution 2) with apparent diffusion coefficient (ADC) maps, T1-weighted in- and opposed-phase imaging, and dynamic T1- weighted imaging before and after injection of contrast agent in the late arterial phase, portal venous phase (60 s), delayed phase (ECA-MRI; 180s) or transitional phase (EOB-MRI; 180 s), and hepatobiliary phase (EOB-MRI; 20 minutes). At both institution 1 and 2, the arterial phase images were obtained either by the acquisition triggered 7 s after arrival of the contrast bolus in the celiac trunk or a multiple arterial phase (MAP) imaging technique. In specific, the MAP images were acquired with an 18 s breath hold 20 s after the contrast media injection, and further reconstructed with a temporal resolution of 3 s. For EOB-MRI, gadoxetate disodium (Primovist®; Bayer Schering Pharma AG) was administered intravenously at 1.0-2.0 ml/s (0.025 mmol/kg of body weight), with an immediately followed 20-30 ml saline flush. For ECA-MRI, gadopentetate dimeglumine (Magnevist®; Bayer Schering Pharma AG) or gadoterate meglumine (Dotarem®; Guerbet) or gadobenate dimeglumine (MultiHance®; Bracco) was administered intravenously at 2.5 ml/s (0.1 mmol/kg of body weight). MRI sequences and parameters are detailed in **Table S1.**

Table S1 MRI sequences and parameters

Sequence	T1-weighted IP and OP imaging	Dynamic T1-weighted 3D GRE	T2-weighted 2D FSE	Diffusion-weighted imaging[†]
**GE Discovery MR 750 3.0 Tesla (16-channel phased-array torsor coil) (institution 1)**				
Repetition time (ms)	150	4.1	6315	9230
Echo time (ms)	2.5/1.3	1.9	78	Minimum
Flip angle (°)	70	15	111	90
Section thickness (mm)	6	2	6	6
Spacing (mm)	2	-	2	2
Matrix size	288×192	512×512	288×244	128×128
Field of view (mm^2)	420×420	380×300	360×280	360×380
Acquisition time (s)	31	15	RG	RG
Fat suppression	No	Yes	Yes	Yes
**GE Discovery MR 750 3.0 Tesla (8-channel body array coil) (institution 2)**				
Repetition time (ms)	NA	4.1	6315	9230
Echo time (ms)	NA	1.9	78	Minimum
Flip angle (°)	NA	15	111	90
Section thickness (mm)	NA	2	6	6
Spacing (mm)	NA	-	2	2
Matrix size	NA	512×512	288×244	128×128
Field of view (mm^2)	NA	380×300	360×280	360×380
Acquisition time (s)	NA	NA	RG	RG
Fat suppression	NA	Yes	Yes	Yes
**GE SIGNA™ Architect 3.0 Tesla (30-channel body anterior coil) (institution 1)**				
Repetition time (ms)	233.8	3.9	2400	5000
Echo time (ms)	2.3/1.1	1.7	85	Minimum
Flip angle (°)	55	15	111	90
Section thickness (mm)	7	3	7	7
Spacing (mm)	2	-	2	2
Matrix size	160×288	320×240	320×192	160×128
Field of view (mm^2)	380×323	380×380	380×304	380×342
Acquisition time (s)	18	15	34	RG
Fat suppression	No	Yes	Yes	Yes
**GE SIGNA™ Premier 3.0 Tesla (30-channel body anterior coil) (institution 1)**				
Repetition time (ms)	146.8	3.2	2200	5000
Echo time (ms)	2.3/1.1	1.4	85	Minimum
Flip angle (°)	55	15	111	90
Section thickness (mm)	7	2.4	7	7
Spacing (mm)	2	-	2	2
Matrix size	320×192	320×240	320×224	120×240
Field of view (mm^2)	342×380	380×380	304×380	380×380
Acquisition time (s)	16	15	47	RG
Fat suppression	No	Yes	Yes	Yes

**Siemens MAGNETOM Skyra 3.0 Tesla (18-channel body array coil) (institution 1)**				
Repetition time (ms)	81	3.95	2160	5600
Echo time (ms)	2.72/1.4	1.92	100	68
Flip angle (°)	70	9	160	90
Section thickness (mm)	6	2.5	6	6
Spacing (mm)	1.8	-	1.8	1.8
Matrix size	352×286	352×256	320×288	100×76
Field of view (mm^2)	400×325	400×296	433×433	380×289
Acquisition time (s)	24	14	36	233
Fat suppression	No	Yes	Yes	Yes
**Siemens TrioTim 3.0 Tesla (8-channel body anterior coil) (institution 1)**				
Repetition time (ms)	181	3.47	2700	5900
Echo time (ms)	2.2/3.67	1.25	95	76
Flip angle (°)	65	9	140	90
Section thickness (mm)	6	2.4	6	6
Spacing (mm)	7.8	-	7.8	7.8
Matrix size	256×131	320×133	320×147	192×154
Field of view (mm^2)	410×269	434×257	442×254	393×393
Acquisition time (s)	18	17	RG	245
Fat suppression	No	Yes	Yes	Yes
**Siemens Avanto 1.5 Tesla (30-channel body anterior coil) (institution 1)**				
Repetition time (ms)	72	5.41	2530	3600
Echo time (ms)	4.92/2.22	2.39	84	88
Flip angle (°)	70	10	150	90
Section thickness (mm)	6	2.5	6	6
Spacing (mm)	7.8	-	7.8	7.8
Matrix size	256×158	320×138	256×187	192×115
Field of view (mm^2)	328×225	382×238	293×251	310×232
Acquisition time (s)	16	15	47	92
Fat suppression	No	Yes	Yes	Yes
**Siemens Avanto 1.5 Tesla (8-channel body anterior coil) (institution 1)**				
Repetition time (ms)	87	5.4	2710	2000
Echo time (ms)	4.92/2.22	2.38	84	72
Flip angle (°)	70	10	150	90
Section thickness (mm)	7.5	2	7.5	7.5
Spacing (mm)	9.75	-	9.75	9.75
Matrix size	256×187	320×131	256×177	192×125
Field of view (mm^2)	308×380	241×407	308×380	308×379
Acquisition time (s)	33	15	27	20
Fat suppression	No	Yes	Yes	Yes
**Philips Ingenia Elition X 3.0 Tesla (16-channel body anterior coil) (institution 1)**				
Repetition time (ms)	164.53	4.20	1883.51	1653.65
Echo time (ms)	2.30/1.15	0.00	90	60.29
Flip angle (°)	50	10	90	90

Section thickness (mm)	6	3	6.8	7
Spacing (mm)	7.5	1.5	8.5	8.5
Matrix size	256×201	344×252	272×78	142×140
Field of view (mm^2)	360×360	380×380	380×380	380×380
Acquisition time (s)	11	13	46	52
Fat suppression	No	Yes	Yes	Yes
**uMR588 1.5 Tesla (6-channel body anterior coil) (institution 1)**				
Repetition time (ms)	117.6	4.2	2600	3350
Echo time (ms)	4.7/2.27	1.88	99.2	77
Flip angle (°)	60	10	90	90
Section thickness (mm)	6.5	2.5	6.5	6.5
Spacing (mm)	1.3	-	1.5	10
Matrix size	256×174	256×154	256×168	128×92
Field of view (mm^2)	320×400	255×400	427×320	320×400
Acquisition time (s)	29	13	39	RG
Fat suppression	No	Yes	Yes	Yes

FSE, fast spin-echo; GRE, gradient recall echo; IP, in-phase; MRI, magnetic resonance imaging; NA, not available; OP, opposed-phase; RG, respiratory gating; 3D, three-dimensional; 2D, two-dimensional.

†Images were acquired under free breath.

Table S2 Characteristics of study patients before PSM and SMDs before and after PSM

Matching variable	Before PSM			SMD§	
	Training cohort (n = 210)	ECA-MRI cohort (n = 661)	*P* value	Before PSM	After PSM
Age (y)†	52.1 ± 11.6	53.0 ± 10.9	0.302	0.078	0.128
Sex (male)	173 (82.4)	581 (87.9)	0.054	0.145	0.063
Cirrhosis	104 (49.5)	360 (54.5)	0.242	0.099	0.029
BCLC stage			<0.001		
0	29 (13.8)	87 (13.2)		0.019	0.014
A	101 (48.1)	437 (66.1)		0.361	0.000
B	34 (16.2)	68 (10.3)		0.160	0.078
C	46 (21.9)	69 (10.4)		0.277	0.058
No. of death	42 (20.0)	216 (32.7)	0.001	0.317	0.012

Unless indicated otherwise, data are the number of patients, with percentages in parentheses. SMD was defined as follows: <0.1, very small differences; 0.1–0.3, small differences; 0.3–0.5, moderate differences; and >0.5, large differences. Group comparison was performed with the Student's *t* test for continuous variable and Chi-square test or Fisher's exact test for categorical variables, as appropriate.

†Data are means ± standard deviations.

§Data are presented as the absolute value of SMD.

BCLC, Barcelona Clinic Liver Cancer; ECA-MRI, extracellular contrast agent-enhanced magnetic resonance imaging; PSM, propensity score matching; SMD, standardized mean difference.

Table S3 Interobserver agreement for the OSASH score

Characteristic	Training cohort (n = 210)	Internal validation cohort (n = 210)	External validation cohort (n = 100)
Incomplete tumor "capsule"	0.42 (0.29-0.55)	0.42 (0.28-0.57)	0.46 (0.24-0.68)
Mosaic architecture	0.40 (0.28-0.53)	0.59 (0.48-0.71)	0.71 (0.56-0.86)
Tumor multiplicity	0.73 (0.64-0.82)	0.63 (0.52-0.74)	0.80 (0.70-0.90)
AFP level	…	…	…
OSASH score†	0.72 (0.64-0.78)	0.66 (0.57-0.73)	0.72 (0.62-0.80)

Unless indicated otherwise, data are κ statistics, with 95% confidence interval in parentheses.

†Data are intraclass correlation coefficients, with 95% confidence interval in parentheses.

Interobserver agreement was assessed by using k statistics or intraclass correlation coefficients, as follows: 0.20 or less, poor agreement; 0.21-0.40, fair agreement; 0.41-0.60, moderate agreement; 0.61-0.80, substantial agreement; and greater than 0.80, almost perfect agreement.

AFP, alpha-fetoprotein.

Table S4 Median OS, 3- and 5-year OS rates, and hazard ratios for BCLC stage subgroups and OSASH score risk subclasses in patients with BCLC stage 0-A and B-C HCC in the training and internal validation cohorts

Variable and risk group	Training cohort (n = 210)						Internal validation cohort (n = 210)					
	No.	Median OS, months (95%CI)	3-year OS rate, % (95%CI)	5-year OS rate, % (95%CI)	Hazard Ratio (95%CI)	*P* value	No.	Median OS, months (95%CI)	3-year OS rate, % (95%CI)	5-year OS rate, % (95%CI)	Hazard Ratio (95%CI)	*P* value
BCLC stage						0.178						0.444
0	29	NA (NA-NA)	100.0 (100.0-100.0)	100.0 (100.0-100.0)	Reference		28	NA (NA-NA)	96.3 (89.4-100.0)	96.3 (89.4-100.0)	Reference	
A	101	NA (NA-NA)	93.3 (88.2-98.7)	89.6 (82.7-97.0)	28.23 (0.02-40050.15)		101	NA (NA-NA)	94.4 (89.7-99.3)	92.7 (87.0-98.6)	2.22 (0.28-17.76)	
BCLC stage						0.185						<0.001
B	34	NA (43.4-NA)	84.0 (71.9-98.2)	52.7 (35.9-77.3)	Reference		40	NA (NA-NA)	85.5 (74.4-98.2)	85.5 (74.4-98.2)	Reference	
C	46	56.4 (26.2-NA)	53.6 (40.3-71.1)	49.4 (35.7-68.4)	1.61 (0.79-3.29)		41	20.7 (13.6-NA)	43.6 (30.7-61.9)	31.2 (19.2-50.5)	7.73 (2.97-20.13)	
BCLC stage 0-A HCC						<0.001						0.034
Low risk	125	NA (NA-NA)	97.5 (94.7-100.0)	94.1 (89.0-99.6)	Reference		120	NA (NA-NA)	96.1 (92.5-99.9)	94.6 (90.0-99.4)	Reference	
High risk	5	27.0 (23.3-NA)	40.0 (13.7-100.0)	40.0 (13.7-100.0)	15.72 (3.87-63.77)		9	NA (NA-NA)	77.8 (54.9-100.0)	77.8 (54.9-100.0)	5.20 (1.01-26.84)	
BCLC stage B-C HCC						0.001						<0.001
Low risk	36	NA (NA-NA)	86.3 (74.5-100.0)	67.5 (50.8-89.7)	Reference		50	NA (NA-NA)	80.6 (70.0-92.9)	74.7 (62.6-89.2)	Reference	
High risk	44	29.7 (23.2-NA)	49.6 (36.3-67.7)	34.3 (21.3-55.4)	3.68 (1.66-8.18)		31	14.0 (11.3-NA)	36.4 (22.5-58.9)	28.7 (15.9-51.5)	5.30 (2.54-11.07)	

BCLC, Barcelona Clinic Liver Cancer; CI, confidence interval; HCC, hepatocellular carcinoma; NA, not available; OS, overall survival.

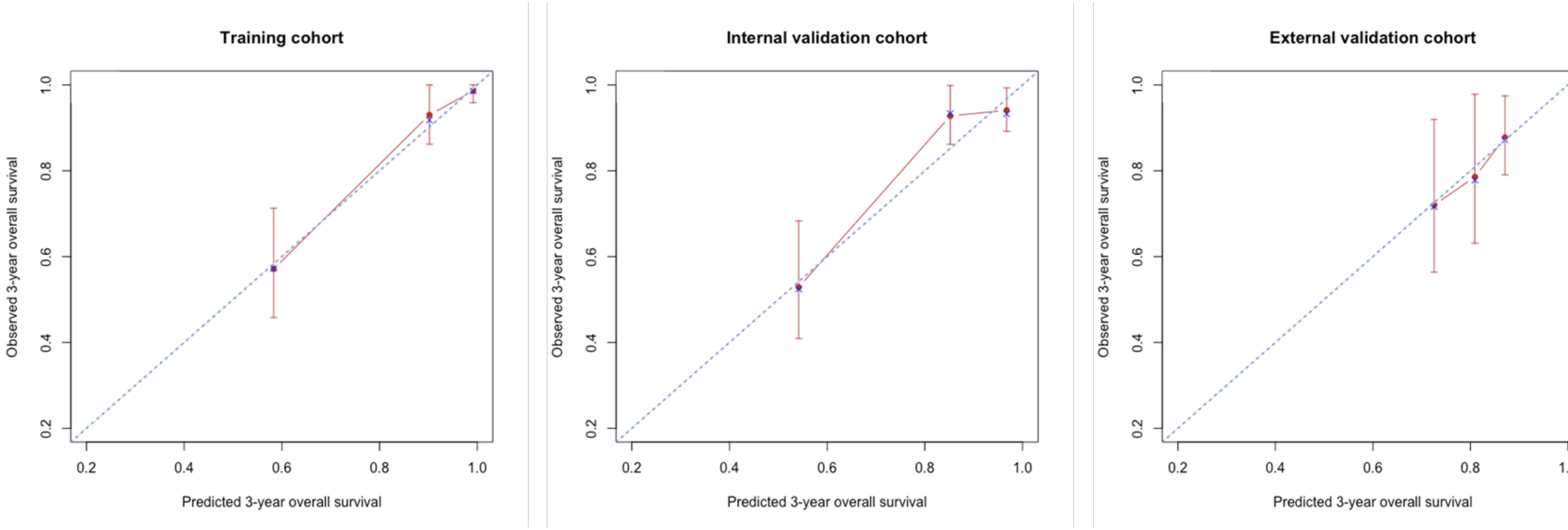

Figure S1 Calibration plots for the OSASH score in predicting 3-year overall survival in all cohorts.

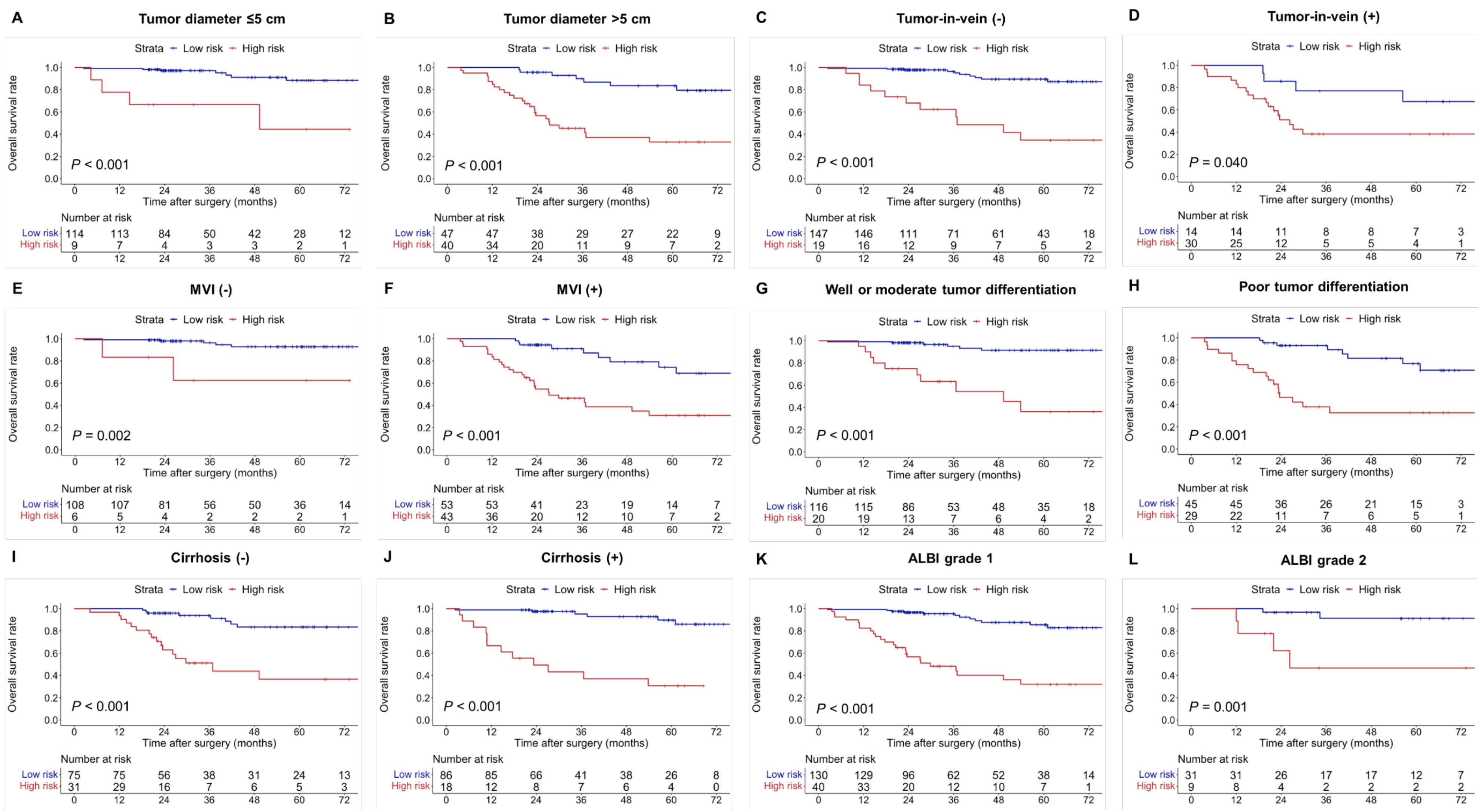

Figure S2 Kaplan-Meier curves demonstrating differences in OS between the OSASH-low and OSASH-high risk patients with HCC in six subgroups in the training cohort. ALBI, albumin-bilirubin; HCC, hepatocellular carcinoma; MVI, microvascular invasion; OS, overall survival.

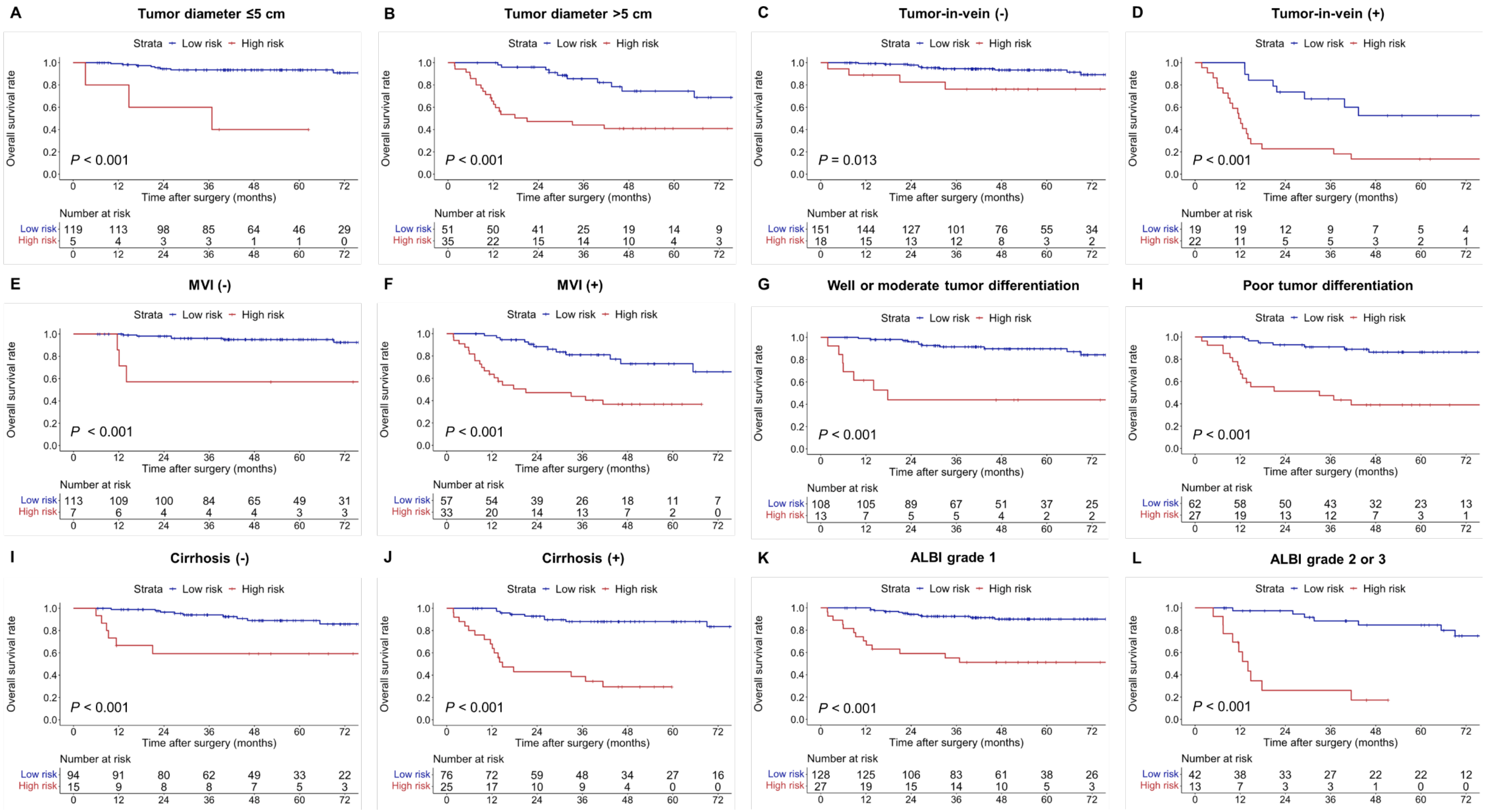

Figure S3 Kaplan-Meier curves demonstrating differences in OS between the OSASH-low and OSASH-high risk patients with HCC in six subgroups in the internal validation cohort. ALBI, albumin-bilirubin; HCC, hepatocellular carcinoma; MVI, microvascular invasion; OS, overall survival.

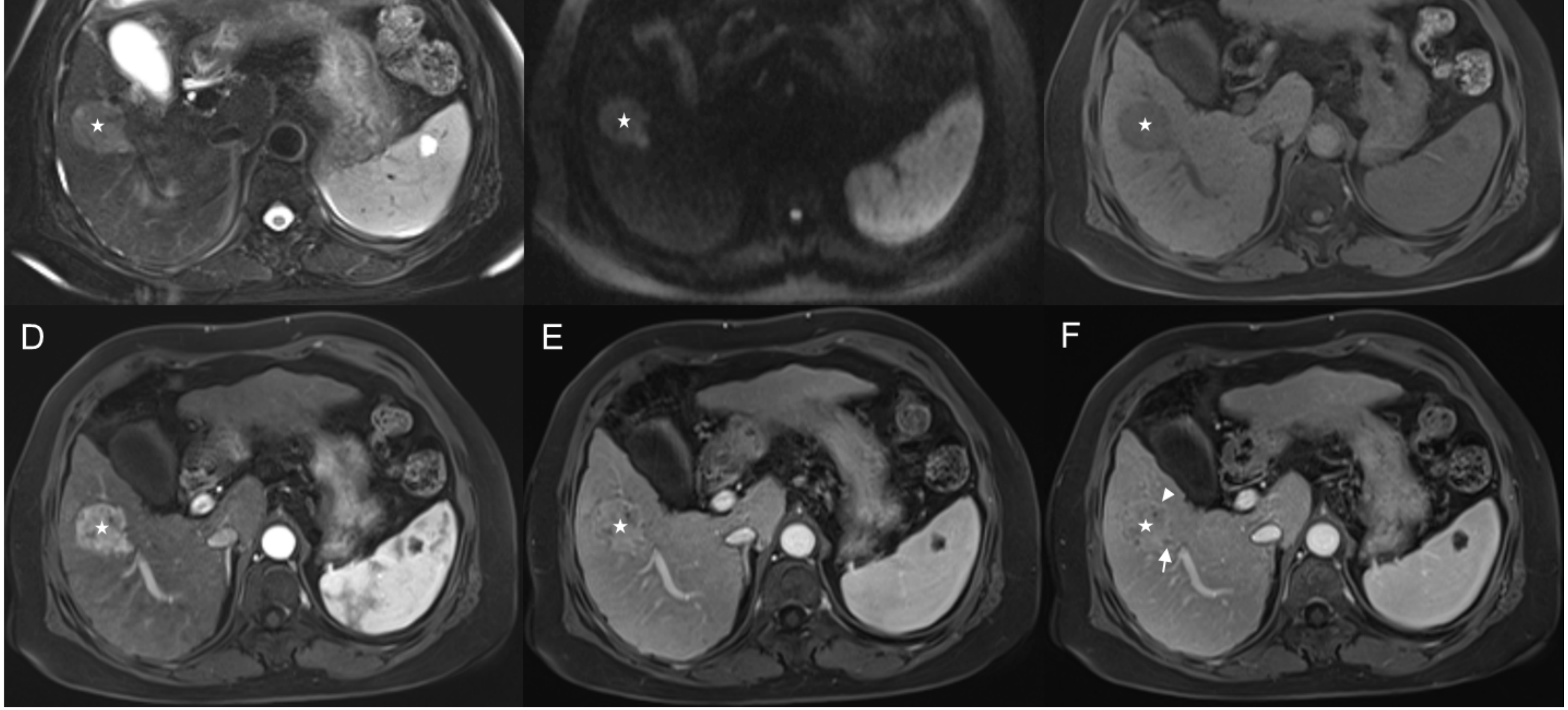

Figure S4 An OSASH-low risk patient with BCLC stage C HCC in the internal validation cohort. Pathologically confirmed HCC in a 64-year-old woman with baseline serum AFP of 78 ng/ml. (**A–F**) Extracellular contrast agent-enhanced MRI demonstrated a 5.7 cm HCC in liver segment V. The tumor (star) showed mild to moderate hyperintensity on (**A**) T2-weighted image, diffusion restriction on (**B**) diffusion-weighted image (b = 1200 s/mm^2), hypointensity on (**C**) precontrast agent-enhanced T1-weighted image, nonrim arterial phase hyperenhancement on (**D**) late arterial phase image, nonsmooth tumor margin on (**E**) portal venous phase, and incomplete tumor “capsule” (arrowhead) and portal vein tumor thrombus (arrow) on (**F**) delayed phase image. This patient was classified as BCLC stage C before surgery. She had one risk factor (incomplete tumor “capsule”) for overall survival and was assigned 20 points, corresponding to the CONMA-low risk group (≤32 points). The patient was alive throughout the follow-up period of 51.1 months. AFP, alpha-fetoprotein; BCLC, Barcelona Clinic Liver Cancer; HCC, hepatocellular carcinoma; MRI, magnetic resonance imaging.
